# Identification of Key Antioxidants of Free, Esterified, and Bound Phenolics in Walnut Kernel and Skin

**DOI:** 10.3390/foods12040825

**Published:** 2023-02-15

**Authors:** Shutian Wu, Runhong Mo, Ruohui Wang, Qingyang Li, Danyu Shen, Yihua Liu

**Affiliations:** 1 Research Institute of Subtropical Forestry, Chinese Academy of Forestry, Fuyang 311400, China; 2 Shanghai Jing’an District Environmental Monitoring Station, Shanghai 200072, China

**Keywords:** walnut kernel, phenolic form, key antioxidant, contribution rate

## Abstract

Walnut is a natural source of antioxidants. Its antioxidant capacity is determined by the distribution and composition of phenolics. The key phenolic antioxidants in various forms (free, esterified, and bound) in walnut kernel (particularly seed skin) are unknown. The phenolic compounds in twelve walnut cultivars were analyzed using ultra-performance liquid chromatography coupled with a triple quadrupole mass spectrometer in this study. A boosted regression tree analysis was used to identify the key antioxidants. Ellagic acid, gallic acid, catechin, ferulic acid, and epicatechin were abundant in the kernel and skin. The majority of phenolic acids were widely distributed in the free, esterified, and bound forms in the kernel but more concentrated in bound phenolics in the skin. The total phenolic levels of the three forms were positively correlated with antioxidant activities (R = 0.76–0.94, *p* < 0.05). Ellagic acid was the most important antioxidant in the kernel, accounting for more than 20%, 40%, and 15% of antioxidants, respectively. Caffeic acid was responsible for up to 25% of free phenolics and 40% of esterified phenolics in the skin. The differences in the antioxidant activity between the cultivars were explained by the total phenolics and key antioxidants. The identification of key antioxidants is critical for new walnut industrial applications and functional food design in food chemistry.

## 1. Introduction

Walnut (*Juglans regia* L.) is a nutritious and delicious tree nut that is widespread in the world [1,2]. Walnuts have long been valued for their contribution to human health and nutritional properties and, in the food industry, due to their high concentration of phenolic compounds [3]. According to their existence forms in the food matrix, phenolics can be classified into soluble-free phenolics, soluble-esterified phenolics, and insoluble-bound phenolics [4,5]. The majority of published papers in recent years have focused on the three phenolic forms of fruits [6,7], vegetables [8], plant leaves [9], and cereal grains [10,11]. Rocchetti et al. investigated free and bound phenolics in five tree nuts and discovered that free phenolics were the most abundant [12]. Significant differences in phenolic forms among walnut, fruits, and grains on walnut kernel (without skin) were discovered in a previous study [13]. However, the phenolics in walnut were not distributed evenly [14]. The phenolic content of the walnut skin (seed coat) was significantly higher than that of the kernel [15]. More attention should be paid to the presence of various phenolic compounds in walnut skin. Because walnut skin is a significant waste product in the food industry, a comprehensive study of walnut kernel and skin can assist us in better understanding phenolic distribution and provide support for effective walnut resource utilization.

In addition to exploring the phenolic profiles of various foods, such as tea [16], fruits [17,18], and cereals [19], many researchers have recently begun to identify the key phenolic antioxidants in these foods [20,21]. Key antioxidant identification is critical for comprehensive food development and functional food design. Some researchers have used correlation analyses to investigate the relationships between different monomeric phenols and antioxidant capacity [20,21]. Khanh-Van et al. used a correlation analysis to investigate the antioxidant activities of sixteen phenolic compounds in black walnut [21]. However, no information was provided about the contributions of specific phenolic compounds to overall antioxidant activities in the walnut. Using this method, it is difficult to quantify the contribution of different monomeric phenols to antioxidant capacity. To investigate the correlations of the distributions of phenolic profiles and antioxidant activities in foods, such as wheat [19] and blackberry [20], some researchers used the boosted regression tree (BRT) method. Previous work has discovered significant positive correlations between total phenolic content (TPC) and antioxidant activity in a single walnut kernel regardless of whether the phenolics were free, esterified, or bound [13]. In this study, we chose twelve common walnut cultivars that were planted in the same producing areas of northern China. The goals were to (1) determine the levels of free, esterified, and bound phenolics in the walnut cultivars; (2) compare the distribution of phenolics between the kernels and skin; and (3) identify the key antioxidant phenolics in three forms and determine contribution rates using BRT. The findings of this study will not only help in the identification of new industrial applications for walnuts (particularly the skin) but will also aid in the breeding of high-quality cultivars.

## 2. Material and Methods

### 2.1. Plant Materials

Twelve walnut cultivars (Luguo 3, Luguo 4, Luguo 5, Luguo 6, Luguo 7, Luguo 8, Luguo 9, Luguo 10, Luguo 11, Luguo12, Li1, and Li2, referred to as L1-L12) were collected at commercial maturity from walnut trees (located in the same orchard in Shanxi province of China). Before analysis, the dried walnuts (dry at 40 °C to moisture content of less than 6%) were shelled to obtain kernels. The oil was extracted from the kernel using petroleum ether. The kernel was then placed in a mortar and slowly immersed in liquid nitrogen. The kernel was immediately poured into vitamin C/water (0.5%, *w*/*v*) after cream appeared on the surface. Sonication was carried out for 1 min, followed by 10 min at room temperature. The kernel and skin were separated via manual removal. The final samples (kernel and skin) were stored at −20 ℃ until analysis.

### 2.2. Chemicals and Reagents

Certified reference standards of all 37 targeted phenolic compounds (purity > 98%) were purchased from Sigma-Aldrich Chemical Co., Ltd. (Shanghai, China). Table S1 contains detailed information on the 37 phenolic compounds. Merck (Hangzhou, China) supplied other solvents, such as HPLC-grade methanol (MeOH), ethanol (EtOH), acetonitrile (ACN), and formic acid (FA).

### 2.3. Extraction of Different Phenolic Forms

The walnut sample (kernel or skin, 1.0 g) was extracted with 50 mL of 70% acetone–water solution in a cold-water bath. The extraction process was repeated three times, and then the combined liquid extracts were flash-evaporated to remove the acetone. After the pH of the liquid extracts was adjusted using 2 M HCl to 2.0, liquid–liquid extraction was carried out using ethyl acetate (three times). The supernatants were evaporated and then redissolved in MS-grade methanol to obtain the free phenolics.

The leftover product and solid residue were simultaneously treated with 2 M NaOH for 30 min in an inert atmosphere. After adjusting the pH to 2 with 2 M HCl, the subsequent operation steps were consistent with those of the treatment process of the free phenolics. The details of the extraction process can be seen in our previous work [13].

### 2.4. Total Phenolic Content

The Folin–Ciocalteu method according to Singleton et al. [22] with slight modification was employed to measure the TPC. We added 1 mL of diluted phenolic extract (free, esterified, or bound) to 0.5 mL of Folin–Ciocalteu reagent. Then, 3 mL of sodium carbonate (Na_2_CO_3_, 75 g/L) was added after 5 min. The mixture was diluted to 10 mL and kept in the dark at 40 °C for 1 h. The TPC was measured at 765 nm, and the concentration is expressed as mg of gallic acid equivalents (GAE)/ g (DW).

### 2.5. UPLC-MS/MS

The ultra-performance liquid chromatography coupled with a triple quadrupole mass spectrometer (UPLC-MS/MS) system consisted of an ultra-high-performance liquid chromatographer (Agilent 1290 Infinity II, Agilent Technologies Inc., San Diego, CA, USA) with a Poroshell 120 EC-C18 column (100 × 2.1 mm, 1.9 μm), coupled with a triple quadrupole mass spectrometer (6460C QQQ, Agilent Technologies Inc., San Diego, CA, USA) equipped with electrospray ionization (ESI). The mobile phase was 0.5% formic acid in water (A) and acetonitrile/methanol (8:2, *v*/*v*, B). The gradient conditions were 0–1 min/15%, 20% B; 12.0 min, 50% B; 15.0 min, 60% B; 17.0–19.0 min, 95% B; and 19.0–20.0 min, 15% B. The MS/MS system was operated using electrospray ionization (ESI) in the negative ion mode with a capillary voltage of 3.0 kV. The ionization source and desolvation temperatures were programmed to 120 ℃ and 500 ℃, respectively. The detailed UPLC-MS/MS parameters can be seen in our previous work [13]. Total ions chromatograph (TIC) and typical chromatograms of phenolic compounds at negative (A) and positive (B) MS ionization could be seen in Figure S1 and Figure S2, respectively.

### 2.6. Antioxidant Activities

The free radical scavenging capacity of DPPH assay was employed to evaluate antioxidant activity and free radical scavenging capacity with some modifications [23]. The diluted phenolic extracts were added to 2 mL of an ethanol solution and mixed with 2 mL of a 10^−4^ mol/L DPPH solution (prepared using ethanol). The incubation was left to stand for 10 min in the darkness at room temperature. The absorption of the sample was observed at 517 nm.

### 2.7. Statistical Analysis

The data are expressed as the mean value ± standard deviation (SD). All experiments were carried out in triplicate. Statistical analyses were carried out using PASW Statistic 18.0 software (SPSS, Chicago, IL, USA), and statistical differences of ANOVA tests were followed by Tukey’s tests at a significance level of *p* < 0.05. A boosted regression tree (BRT) analysis was performed according to Podio et al. [24] using R software (version 4.0.3, https://www.R-project.org/, accessed on 8 January 2023).

## 3. Results

### 3.1. TPCs in Free, Esterified, and Bound Forms in Kernel and Skin

Table 1 shows the TPC levels in the walnut kernel and skin. The mean TPC content of the walnut skin (256.78 mg/g) was significantly higher than that of the walnut kernel (21.49 mg/g). The TPC values obtained from the various walnut cultivars differed significantly (*p* < 0.05), with the highest variance being 42.42%. The cultivar “L12” had the highest TPC value in the kernel (35.43 mg/g), and the cultivar “L11” had the highest TPC value in the skin (385.25 mg/g).

Walnut phenolics were found primarily in the free form in both the kernel and the skin, with only a trace amount found in the esterified and bound forms. The free form in the kernel accounted for 14.42 mg/g of the TPCs, followed by the esterified form (4.22 mg/g) and the bound form (2.86 mg/g). The TPCs in the skin had mean contents of 171.84 mg/g, 54.75 mg/g, and 30.2 mg/g in free, esterified, and bound forms, respectively.

### 3.2. Antioxidant Activities of Total Phenolics in Kernel and Skin

The antioxidant activities of the walnut kernels from different cultivars differed significantly (*p* < 0.05). The differences in bound phenolics were up to 28.3%, higher than those in free (12.1%) and esterified (11.5%) phenolics.

The average IC_50_ (half maximal inhibitory concentration) values of the free phenolics (16.53 μg/mL) in the kernel were clearly lower than those of the esterified (36.32 μg/mL) and bound phenolics (84.20 μg/mL), indicating that the free phenolics had the highest antioxidant activity (Table 1). Similarly, the antioxidant activity of the free phenolics in the walnut skin (IC_50_ values: 7.21 μg/mL) was higher than that of the esterified (IC_50_ values: 27.30 μg/mL) and bound forms (IC_50_ values: 48.96 μg/mL). Furthermore, the antioxidant activities of the three phenolic forms found in the skin were all higher than those of the corresponding forms found in the kernel.

### 3.3. Content of Free, Esterified, and Bound Phenolic Compounds in Kernel and Skin

The skin contained far more phenolic compounds than the kernel, including ellagic acid, gallic acid, ferulic acid, catechin, epicatechin, epicatechin gallate, quercetin-7-O-*β*-D-glucoside, dihydroquercetin, and procyanidin B2 (Table 2). Gallic acid (275.24 μg/g), catechin (223.73 μg/g), epicatechin gallate (25.4 μg/g), and quercetin-7-O-*β*-D-glucoside (12.67 μg/g) were all more than 15 times higher in the skin than in the kernel (17.33 μg/g, 8.04 μg/g, 0.72 μg/g, and 0.47 μg/g, respectively).

The highest concentrations of ellagic acid were found in the kernel (109.88 μg/g) and the skin (1666.90 μg/g). Only *p*-hydroxybenzoic acid (4.86 μg/g) in the kernel outperformed that in the skin (1.40 μg/g) among the detected compounds. Ellagic acid, gallic acid, and vanillic acid were free phenolics in the kernel, while the bound phenolics included ferulic acid, caffeic acid, sinapic acid, and protocatechuic acid. The majority of the flavonoid compounds were found to be more abundant in free phenolics than in esterified and bound phenolics. Most phenolic acids, with the exception of ellagic acid and chlorogenic acid, had high levels of bound phenolics in the skin. Fourteen of the sixteen flavan-3-ol compounds identified contained significant amounts of free phenolics, particularly catechin (93.31 μg/g), epicatechin (71.59 μg/g), quercetin-7-O-*β*-D-glucoside (11.25 μg/g), and dihydroquercetin (21.23 μg/g).

### 3.4. Antioxidant Activity of Monomer Phenolic Compounds

Based on the DPPH radical scavenging capacity results (Table 3), it was discovered that quercetin, caffeic acid, catechin gallate, gallocatechin gallate, syringic acid, and epigallocatechin gallate had strong antioxidant activity with IC_50_ values of 1.19–1.49 μg/mL, whereas no antioxidant activity (extremely low) was detected in cumallic acid, dendrobine, lycorine, juglone, cinnamic acid, or *p*-hydroxybenzoic acid. To compare the antioxidant activities of the phenolic compounds, Trolox was used as a positive control (IC_50_: 5.59 μg/mL).

In terms of antioxidant activity, 18 phenolic compounds outperformed Trolox. The increase in these phenolic compounds over Trolox ranged from 1.21- to 4.70-fold. Quercetin had the highest total antioxidant capacity (4.70 times that of Trolox), followed by caffeic acid (4.4) and catechin gallate (4.3). Except for the compounds with no antioxidant activity (extremely low), four phenolic compounds had a lower antioxidant activity than Trolox, namely, catechin, vanillic acid, chlorogenic acid, and *p*-coumaric acid.

## 4. Discussion

Free phenolics were found in high concentrations not only in the kernel but also in the skin of the walnut. The highest concentrations of ellagic acid were found not only in the kernel (62.9%) but also in the skin (68.0%). However, there are some distinctions between the other major phenolic compounds. The most abundant phenolic compounds in the kernel were gallic acid, catechin, ferulic acid, epicatechin, and protocatechuic acid, while gallic acid, protocatechuic acid, catechin, and sinapic acid were abundant in the skin. Vanillic acid, quercetin-3-O-rutinose, and dendrobine were discovered only in the kernel and not in the skin, whereas kaempferol-3-O-glucoside, vitexin, and luteolin were discovered only in the skin.

**Figure 1:**
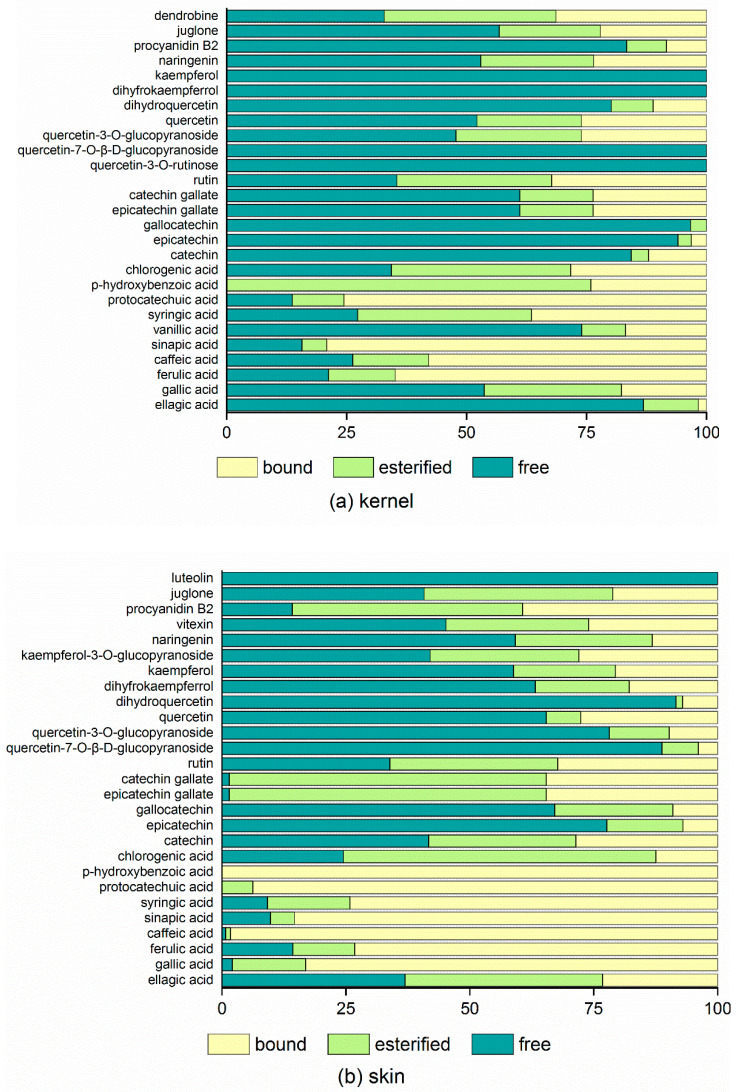
A comparison of phenolic compounds in (**a**) kernel and (**b**) skin of walnut. Note: the horizontal axis indicates percentages of three phenolic forms (free, esterified, and bound); the vertical axis indicates phenolic components.

Specific monomeric phenols found only in the kernel or skin of Brazil nut [25] and pistachio [26] have also been reported. Pistachio skin contained more than 80.0% protocatechuic acid and catechin, but no procyanidins, epicatechin, or epicatechin gallate could be found in pistachio kernel [26]. With the exception of ellagic acid (free), *p*-hydroxybenzoic acid (ester), and sinapic acid (bound), the majority of phenolic acids were widely distributed in the three forms in the walnut kernel (Figure 1a). Phenolic acids in the bound form, particularly *p*-hydroxybenzoic acid, protocatechuic acid, and caffeic acid, were found in high concentrations in the walnut skin (Figure 1b). Previous research found that bound phenolics were more likely than free phenolics to be combined with polysaccharide, pectin, and cellulose in the cell wall structure [27]. The presence of high levels of fiber in the skin might be the primary cause of the emergence of phenolic acids in the bound form. The flavonoid compounds found in higher concentrations in free phenolics in both the kernel and skin included epicatechin, gallacatechin, naringenin, dihydroquercetin, and procyanidin B2. Thirteen of the seventeen phenolic compounds contained more than 50% free phenolics, particularly in the kernel. Furthermore, quercetin-3-O-rutinose, quercetin-7-O-*β*-D-glucoside, dihyfrokaempferrol, and kaempferol were found only in free phenolics in the kernel, whereas luteolin was found only in free phenolics in the skin. Interestingly, catechin gallate and epicatechin gallate were discovered to be concentrated in free phenolics in the kernel, whereas esterified phenolics were found to be more prevalent in the skin.

**Figure 2:**
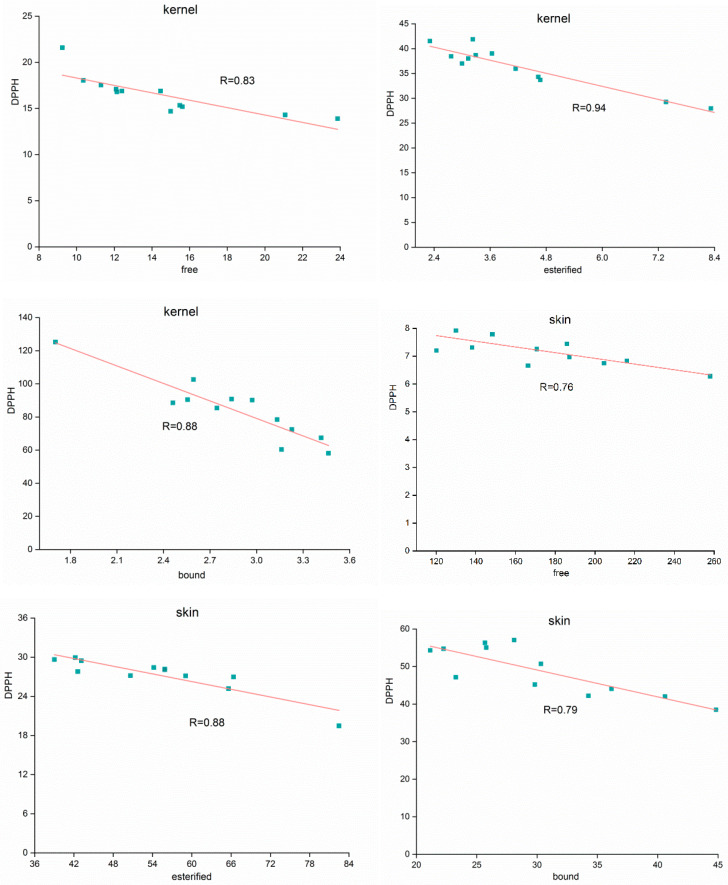
The linear correlations between antioxidant activity assays determined using DPPH scavenging methods and TPC content in free, esterified, and bound forms in kernel and skin of walnut.

**Figure 3:**
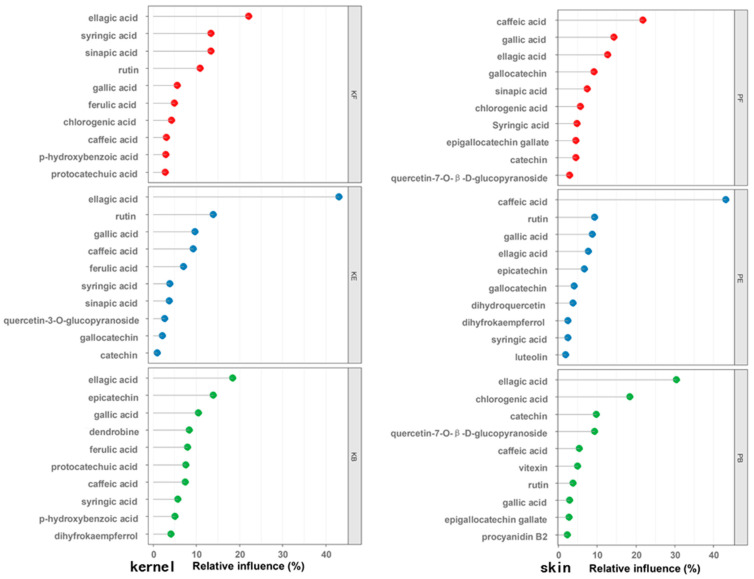
The influence rates of key phenolic compounds to antioxidant capacity in kernel and skin. Note: “KF, KE, KB”, free, esterified and bound phenolics in kernel, respectively. “PF, PE, PB”, free, esterified, and bound phenolics in skin, respectively. The horizontal axis indicates percentages of relative influence; the vertical axis indicates phenolic components.

The TPC levels in the free, esterified, and bound phenolics in the kernel were found to have positive linear correlations with antioxidant activities, determined using DPPH scavenging methods, with correlation coefficients ranging from 0.83 to 0.94 (details can be seen in Figure 2). In addition, the coefficients of correlation found between the free, esterified, and bound phenolics and antioxidant activities in the skin were 0.76, 0.88, and 0.79, respectively. Other researchers [28,29] found similar results regarding a good correlation between the TPC and antioxidant activity, but few studies looked further for the key antioxidant phenolics and compared the contribution of single compounds to antioxidant activity. Khanh-Van et al. discovered ten phenolic compounds in black walnut with high antioxidant activities, with penta-O-galloyl-*β*-D-glucose having the highest antioxidant activity [21]. Surprisingly, a cultivar containing penta-O-galloyl-*β*-D-glucose had low total antioxidant activity. Because of the diverse range of phenolics found in different cultivars, antioxidant activities are likely to be influenced not only by the nature of the compound but also by its content (dosage effect). Our findings (Table 2 and Table 3) show that quercetin, caffeic acid, and catechin gallate had high antioxidant activities but a low content in the walnut, whereas gallic acid and ellagic acid had a high content but low antioxidant activities. Furthermore, a correlation analysis revealed that the majority of monomer phenolics were not significantly associated with antioxidant activities (*p* < 0.05). Thus, in our study, we used BRT, a relatively new assembly method [20], to determine the contribution of monomer compounds to antioxidant activity. The influence rates of major phenolic compounds on antioxidant activities were quantified using BRT (Figure 3). Ellagic acid played a significant role in the free, esterified, and bound forms in the kernel, accounting for more than 20%, 40%, and 15%, respectively. Ellagic acid gut-microbiota-derived metabolites (urolithins) are better absorbed and are responsible for antioxidant activities and health benefits [30]. Thus, ellagic acid could be considered a unique feature of walnut compared with other nuts. Furthermore, syringic acid and sinapic acid (free), rutin and gallic acid (esterified), and epicatechin and gallic acid (bound) all had a significant effect on antioxidant activities. However, caffeic acid, rather than ellagic acid (the most abundant compound), was the most important contributor to antioxidant activities in the skin, contributing up to 25% and 40% to free and esterified phenolics, respectively. Despite the fact that the average caffeic acid content was one-tenth that of ellagic acid, gallic acid and ellagic acid (free), rutin and gallic acid (esterified), and ellagic acid and chlorogenic acid (bound) were all significant phenolic contributors to antioxidant activity. In addition, despite their low concentrations, epigallocatechin gallate and gallocatechin played important roles in antioxidant activity, particularly in the walnut skin. Paiva et al. found that esterified catechin (epigallocatechin-3-gallate, epicatechin-3-gallate) content was associated with higher antioxidant activity [31].

We also discovered that differences in key phenolic antioxidants influenced the antioxidant activity of the different cultivars. In the bound form, cultivars “L7” and “L12”, in particular, demonstrated significantly high antioxidant activities (Table 1). In the two cultivars, the total content of key antioxidants (ellagic acid, gallic acid, and epicatechin) in the bound form in the skin was higher than in the others. Moreover, cultivar “L12” (which had the highest antioxidant activity) contained high levels of key antioxidants (ellagic acid, sinapic acid, syringic acid, and rutin) in the free form in the kernel, as well as the esterified forms of ellagic acid, gallic acid, caffeic acid, and ferulic acid, respectively. In turn, there were no significant differences (*p* > 0.05) between the cultivars in the antioxidant activities of the phenolics in the free (6.3–8.1 μg/mL) and esterified forms (19.5–29.9 μg/mL) in the skins. Despite the significant associations between the TPCs and antioxidant activities, the lack of significant differences in antioxidant activities was primarily due to the high absolute content of key antioxidants in the walnut skin.

## 5. Conclusions

The current study investigated the distribution of phenolic profiles and the key antioxidants in walnut kernels. Both the kernel and the skin contained the majority of the phenolics in their free form. Whole walnut kernels contained significant amounts of ellagic acid, gallic acid, catechin, ferulic acid, epicatechin, and syringic acid. Vanillic acid, quercetin-3-O-rutinose, and dendrobine were only found in the kernel, while kaempferol-3-O-glucoside, vitexin, and luteolin were only found in the skin. The TPCs and antioxidant activities were found to be positively correlated in all three forms. In free, esterified, and bound forms, ellagic acid was the most important phenolic antioxidant, accounting for more than 20%, 40%, and 15%, respectively. Caffeic acid was discovered to be the most powerful phenolic antioxidant in the skin’s free and esterified phenolics, while ellagic acid was discovered to be the most important in the bound phenolics. The difference in the antioxidant activity between the walnut cultivars may be explained by the content of the TPCs and the key phenolic antioxidants.

Our findings provide detailed information on the phenolic distribution in walnut kernels, as well as confirmation that the skin is a good natural raw material for antioxidant substances, with a high exploitation potential. The systematic identification of phenolic profiles and distributions in the kernel and skin may also aid in fully excavating the food matrix for the development of functional food formulations.

In our next study, we will look into verifying the antioxidant capacity of these key phenolic antioxidants in additional walnut cultivar samples. Furthermore, it is worth investigating whether the BRT model or other models can be used to investigate the contribution of different phenolics to other biological activities.

## Figures and Tables

**Table 1:** The contents (mg/g dry weight) and DPPH scavenging capacity (μg/mL) of total phenolics in kernels and skins of 12 walnut cultivars.

Cultivar		Kernel			Skin	
	Free	Ester	Bound	Free	Ester	Bound
Total phenolics						
L1	10.36 ± 1.02 ^cd^	3.29 ± 0.21 ^bc^	3.13 ± 0.34 ^b^	215.97 ± 4.51 ^ab^	65.62 ± 0.46 ^b^	36.17 ± 0.10 ^b^
L2	14.46 ± 1.21 ^bc^	4.63 ± 0.41 ^b^	2.84 ± 0.54 ^b^	120.24 ± 5.16 ^c^	39.02 ± 0.23 ^c^	22.26 ± 0.09 ^d^
L3	9.24 ± 0.72 ^d^	4.15 ± 0.12 ^bc^	3.23 ± 0.12 ^ab^	170.75 ± 4.98 ^b^	54.19 ± 0.34 ^bc^	23.27 ± 0.04 ^d^
L4	14.99 ± 0.54 ^b^	4.67 ± 0.18 ^b^	2.97 ± 0.06 ^b^	166.27 ± 3.21 ^bc^	55.88 ± 1.21 ^bc^	25.79 ± 0.18 ^d^
L5	12.09 ± 1.39 ^c^	3.00 ± 0.59 ^c^	2.74 ± 0.14 ^c^	185.82 ± 8.15 ^b^	59.06 ± 1.58 ^bc^	30.33 ± 0.11 ^b^
L6	12.14 ± 0.57 ^c^	3.13 ± 0.34 ^c^	3.42 ± 0.13 ^a^	148.35 ± 6.18 ^c^	50.63 ± 0.49 ^c^	34.26 ± 0.21 ^b^
L7	15.61 ± 1.28 ^b^	7.36 ± 0.28 ^a^	3.46 ± 0.19 ^a^	138.17 ± 1.21 ^c^	43.14 ± 0.39 ^c^	25.69 ± 0.12 ^cd^
L8	11.29 ± 0.67 ^cd^	2.31 ± 0.26 ^d^	2.46 ± 0.22 ^c^	187.05 ± 0.39 ^b^	66.37 ± 0.44 ^b^	40.61 ± 0.18 ^a^
L9	12.41 ± 0.52 ^c^	3.23 ± 0.34 ^c^	2.59 ± 0.15 ^bc^	204.56 ± 5.14 ^ab^	55.88 ± 0.26 ^bc^	28.11 ± 0.06 ^c^
L10	15.48 ± 0.42 ^b^	3.64 ± 0.32 ^bc^	2.56 ± 0.34 ^bc^	136.95 ± 2.15 ^c^	42.20 ± 0.38 ^c^	29.82 ± 0.13 ^bc^
L11	21.08 ± 1.21 ^a^	2.77 ± 0.29 ^cd^	1.70 ± 0.10 ^d^	257.92 ± 2.98 ^a^	82.48 ± 0.88 ^a^	44.85 ± 0.19 ^a^
L12	23.86 ± 2.15 ^a^	8.40 ± 0.22 ^a^	3.16 ± 0.22 ^b^	130.02 ± 1.56 ^c^	42.58 ± 0.21 ^c^	21.15 ± 0.09 ^d^
CV	28.60%	42.42%	16.48%	12.12%	11.51%	21.29%
Antioxidant activity (DPPH)						
L1	18.05 ± 0.10 ^ab^	38.69 ± 0.14 ^ab^	78.45 ± 1.21 ^cd^	6.84 ± 0.06 ^a^	25.19 ± 0.54 ^a^	44.08 ± 0.34 ^b^
L2	16.90 ± 0.13 ^b^	34.32 ± 0.18 ^bc^	90.85 ± 0.69 ^bc^	7.20 ± 0.10 ^a^	29.66 ± 0.31 ^a^	54.74 ± 0.27 ^ab^
L3	21.60 ± 0.03 ^a^	35.98 ± 0.45 ^bc^	72.54 ± 0.48 ^cd^	7.26 ± 0.16 ^a^	28.42 ± 0.24 ^a^	47.16 ± 0.39 ^b^
L4	14.70 ± 0.12 ^bc^	33.70 ± 0.37 ^bc^	90.18 ± 1.01 ^bc^	6.66 ± 0.34 ^a^	28.09 ± 0.36 ^a^	55.05 ± 0.16 ^ab^
L5	17.10 ± 0.14 ^ab^	37.01 ± 0.26 ^b^	85.45 ± 0.79 ^c^	7.44 ± 0.12 ^a^	27.15 ± 0.15 ^a^	50.74 ± 0.30 ^ab^
L6	16.81 ± 0.24 ^b^	38.02 ± 0.29 ^ab^	67.48 ± 0.66 ^d^	7.78 ± 0.16 ^a^	27.18 ± 0.43 ^a^	42.23 ± 0.57 ^b^
L7	15.20 ± 0.15 ^bc^	29.26 ± 0.49 ^c^	58.14 ± 0.42 ^d^	7.32 ± 0.12 ^a^	29.46 ± 0.22 ^a^	56.35 ± 0.19 ^a^
L8	17.55 ± 0.22 ^ab^	41.54 ± 0.46 ^a^	88.54 ± 1.02 ^c^	6.96 ± 0.15 ^a^	26.98 ± 0.26 ^a^	42.05 ± 0.44 ^b^
L9	16.90 ± 0.15 ^b^	41.88 ± 0.39 ^a^	102.62 ± 1.64 ^b^	6.75 ± 0.06 ^a^	28.17 ± 0.19 ^a^	57.07 ± 0.72 ^a^
L10	15.35 ± 0.09 ^bc^	39.03 ± 0.24 ^ab^	90.52 ± 0.38 ^c^	8.10 ± 0.13 ^a^	29.94 ± 0.33 ^a^	45.21 ± 0.36 ^b^
L11	14.30 ± 0.25 ^c^	38.45 ± 0.31 ^ab^	125.25 ± 1.59 ^a^	6.28 ± 0.08 ^a^	19.49 ± 0.41 ^a^	38.50 ± 0.21 ^c^
L12	13.90 ± 0.03 ^c^	27.97 ± 0.66 ^c^	60.42 ± 0.44 ^d^	7.93 ± 0.04 ^a^	27.80 ± 0.08 ^a^	54.33 ± 0.44 ^ab^

Note: data are expressed as mean ± standard deviation (*n* = 3). The values with different lowercase letters (a,b,c,d) are significantly different at *p* < 0.05 within each column.

**Table 2:** The range of free, esterified, and bound phenolic contents (μg/g dry weight) in kernel and skin.

		Kernel				Skin		
Phenolic Compounds	Free	Esterified	Bound	Total	Free	Esterified	Bound	Total
ellagic acid	56.49–164.95 (95.45)	5.83–28.10 (12.57)	0.82–5.58 (1.86)	109.88	448.15–929.34 (616.44)	600.99–724.70 (663.72)	254.32–602.69 (386.74)	1666.90
gallic acid	4.43–21.05 (9.30)	2.48–9.51 (4.96)	0.49–8.02 (3.07)	17.33	3.85–10.07 (5.69)	24.98–64.47 (40.76)	97.42–354.13 (228.79)	275.24
ferulic acid	0.44–2.90 (1.62)	0.56–2.35 (1.06)	2.59–8.18 (4.95)	7.63	1.08–9.41 (4.63)	2.50–7.32 (4.05)	15.22–45.72 (23.71)	32.39
caffeic acid	nd-0.55 (0.35)	0.03–0.15 (0.09)	0.15–0.48 (0.33)	0.57	0.01–0.08 (0.03)	0.01–0.09 (0.04)	1.81–17.14 (4.02)	4.09
sinapic acid	nd-1.42 (0.49)	nd-0.44 (0.16)	1.05–6.04 (2.47)	3.12	nd-1.87 (0.42)	nd-1.01 (0.21)	1.10–5.87 (3.68)	4.31
vanillic acid	nd-1.82 (0.57)	nd-0.55 (0.07)	nd-0.88 (0.13)	0.77	nd	nd	nd	/
syringic acid	0.28–1.96 (1.35)	0.17–6.15 (1.26)	0.43–3.72 (1.27)	3.48	0.21–2.82 (1.14)	0.34–4.29 (2.07)	2.74–21.63 (9.22)	12.43
protocatechuic acid	nd-1.14 (0.37)	0.01–1.26 (0.29)	0.31–10.68 (2.04)	2.7	nd	Nd-1.20 (0.34)	2.33–9.01 (5.11)	5.45
cinnamic acid	nd	nd	nd	/	nd	nd	nd	/
*p*-hydroxybenzoic acid	nd	1.65–6.23 (3.69)	0–3.31 (1.17)	4.86	nd	nd	0–4.15 (1.40)	1.40
chlorogenic acid	0.28–0.64 (0.54)	0.28–0.51 (0.37)	0.28–0.29 (0.28)	0.99	0.72–2.02 (1.12)	1.26–5.83 (2.88)	0.56–0.58 (0.57)	4.57
*p*-coumaric acid	nd	nd	nd	/	nd	nd	0–0.889	/
(+)-catechin	3.15–15.19 (6.78)	0.13–0.61 (0.29)	0.16–5.80 (0.97)	8.04	41.10–156.48 (93.31)	38.48–98.66 (66.53)	14.00–95.40 (63.89)	223.73
epicatechin	2.78–12.01 (5.70)	0.12–0.25 (0.17)	0.12–0.51 (0.19)	6.06	31.17–128.58 (71.59)	4.88–28.23 (14.14)	1.80–21.67 (6.45)	92.18
(-)-gallocatechin	nd-0.67 (0.29)	nd-0.02 (0.01)	nd	0.3	nd-2.79 (1.41)	nd-1.17 (0.50)	nd-0.93 (0.19)	2.10
epigallocatechin gallate	nd	nd	nd	/	nd	nd	nd	/
(-)-gallocatechin gallate	nd	nd	nd	/	nd	nd	nd	/
(-)-epicatechin gallate	0.09–1.14 (0.44)	0.09–0.19 (0.11)	0.09–0.54 (0.17)	0.72	0.27–0.51 (0.38)	4.31–36.62 (16.24)	1.82–22.06 (8.78)	25.4
(-)-epigallocatechin	nd	nd	nd	/	nd	nd	nd	/
catechin gallate	0.09–1.14 (0.44)	0.09–0.19 (0.11)	0.09–0.54 (0.17)	0.72	0.27–0.51 (0.38)	4.31–36.62 (16.24)	1.82–22.06 (8.78)	25.4
rutin	0.08–0.12 (0.11)	0.08–0.11 (0.10)	0.07–0.13 (0.10)	0.31	0.15–0.37 (0.21)	0.16–0.26 (0.21)	0.17–0.24 (0.20)	0.62
quercetin-3-O-rutinose	0.13–0.17 (0.14)	nd	nd	0.14	nd	nd	nd	/
quercetin-7-O-*β*-D-glucoside	nd-1.22 (0.47)	nd	nd	0.47	0.04–18.14 (11.25)	0.34–2.28 (0.93)	nd-1.82 (0.49)	12.67
quercetin-3-O-glucoside	0.23–0.77 (0.44)	0.23–0.25 (0.24)	0.23–0.26 (0.24)	0.92	4.67–9.82 (6.99)	0.60–1.62 (1.08)	0.56–1.22 (0.87)	8.94
quercetin	0.05–0.20 (0.12)	0.05–0.06 (0.05)	0.05–0.08 (0.06)	0.23	0.63–2.54 (1.42)	0.13–0.17 (0.15)	0.31–0.81 (0.60)	2.17
dihydroquercetin	0.11–3.78 (1.01)	0.11–0.14 (0.11)	0.11–0.23 (0.14)	1.26	7.27–46.33 (21.23)	0.24–0.67 (0.31)	0.22–12.21 (1.63)	23.17
dihyfrokaempferrol	0.13–0.75 (0.46)	nd	nd	0.74	0.29–5.96 (1.17)	0.26–0.54 (0.35)	0.25–0.45 (0.33)	1.85
kaempferol	0.03–0.06 (0.04)	nd	nd	0.04	0.07–0.48 (0.20)	0.07–0.09 (0.07)	0.07–0.09 (0.07)	0.34
kaempferol-3-O-glucoside	nd	nd	nd	/	0.14–0.69 (0.21)	0.13–0.21 (0.15)	0.14–0.16 (0.14)	0.50
naringenin	0.08–0.34 (0.18)	0.08–0.10 (0.08)	0.08–0.09 (0.08)	0.34	0.16–4.44 (0.90)	0.17–2.38 (0.42)	0.16–0.29 (0.20)	1.52
vitexin	nd	nd	nd	/	0.19–0.55 (0.33)	0.19–0.33 (0.21)	0.19–0.21 (0.19)	0.73
procyanidin B_2_	nd-0.70 (0.10)	nd-0.03 (0.01)	nd-0.01 (0.01)	0.12	0.31–9.09 (2.67)	3.32–17.83 (8.76)	nd-17.86 (7.41)	18.84
juglone	0.09–1.58 (0.54)	0.08–0.73 (0.20)	0.08–0.48 (0.21)	0.95	0.17–3.20 (1.06)	0.18–2.51 (0.99)	0.34–0.95 (0.55)	2.60
dendrobine	0.61–0.89 (0.67)	0.61–1.13 (0.73)	0.59–0.74 (0.64)	2.04	nd	nd	nd	/
cumallic acid	nd	nd	nd	/	nd	nd	nd	/
lycorine	nd	nd	nd	/	nd	nd	nd	/
luteolin	nd	nd	nd	/	0.13–0.55 (0.29)	nd	nd	0.29

Note: “nd”, not detected. “/”, no data. All experiments were carried out in three repetitions. Data in parentheses are the mean values.

**Table 3:** The DPPH scavenging capacity of monomer phenolic compounds.

Phenolic Compounds	DPPH (μg/mL)	Antioxidant Capacity	Classification
Trolox	5.59 ± 0.04	1.00	/
antioxidant capacity higher than Trolox			
quercetin	1.19 ± 0.04	4.70	H
caffeic acid	1.27 ± 0.02	4.40	H
catechin gallate	1.30 ± 0.01	4.30	H
gallocatechin gallate	1.47 ± 0.06	3.80	H
syringic acid	1.49 ± 0.01	3.75	H
epigallocatechin gallate	1.49 ± 0.05	3.75	H
epicatechin gallate	1.77 ± 0.02	3.16	M
gallocatechin	1.83 ± 0.10	3.05	M
procyanidin B_2_	2.04 ± 0.06	2.74	M
epiallocatechin	2.14 ± 0.10	2.61	M
gallic acid	2.24 ± 0.02	2.50	M
ellagic acid	2.29 ± 0.09	2.44	M
protocatechuic acid	2.59 ± 0.12	2.16	M
epicatechin	2.60 ± 0.08	2.15	M
sinapic acid	2.87 ± 0.03	1.95	M
kaempferol	3.11 ± 0.09	1.80	M
rutin	3.89 ± 0.05	1.44	M
ferulic acid	4.62 ± 0.11	1.21	M
antioxidant capacity lower than Trolox			
catechin	5.84 ± 0.02	0.96	L
vanillic acid	7.99 ± 0.05	0.70	L
chlorogenic acid	12.0 ± 0.04	0.46	L
*p*-coumaric acid	18.63± 0.03	0.30	L
cumallic acid	nd	/	nd
dendrobine	nd	/	nd
lycorine	nd	/	nd
juglone	nd	/	nd
cinnamic acid	nd	/	nd
*p*-hydroxybenzoic acid	nd	/	nd

Note: “nd”, not detected. “/”, no data. Data are expressed as mean ± standard deviation (*n* = 3). “H”, High; “M”, Middle; “L”, Low.

## Data Availability

Data is contained within the article.

## Supplementary Materials

The following supporting information can be downloaded at: https://www.mdpi.com/article/10.3390/foods12040825/s1, Figure S1: Total ions chromatograph (TIC) obtained at negative (A) and positive (B) MS ionization for standards of phenolic compounds; Figure S2: Typical chromatograms obtained at negative (A) and positive (B) MS ionization for standards of phenolic compounds; Table S1: Mass spectrometry parameters of 37 phenolic compounds for multiple reactions monitoring (MRM).

## References

[1] Abdallah I.B., Tlili N., Martinez-Force E., Rubio AG P., Perez-Camino M.C., Albouchi A., Boukhchina S. (2015). Content of carotenoids, tocopherols, sterols, triterpenic and aliphatic alcohols, and volatile compounds in six walnuts (*Juglans regia* L.) varieties. Food Chem..

[2] Hama J.R., Omer R.A., Rashid R.S.M., Mohammad N.-E.-A., Thoss V. (2016). The Diversity of Phenolic Compounds along Defatted Kernel, Green Husk and Leaves of Walnut (*Juglans regia* L.). Anal. Chem. Lett..

[3] Liu M., Li C., Cao C., Wang L., Li X., Che J., Yang H., Zhang X., Zhao H., He E. (2021). Walnut Fruit Processing Equipment: Academic Insights and Perspectives. Food Eng. Rev..

[4] Lou X.M., Xu H.D., Hanna M., Yuan L. (2020). Identification and quantification of free, esterified, glycosylated and insoluble-bound phenolic compounds in hawthorn berry fruit (*Crataegus pinnatifida*) and antioxidant activity evaluation. Lwt-Food Sci. Technol..

[5] de Camargo A.C., Concepción Alvarez A., Arias-Santé M.F., Oyarzún J.E., Andia M.E., Uribe S., Núñez Pizarro P., Bustos S.M., Schwember A.R., Shahidi F. (2022). Soluble Free, Esterified and Insoluble-Bound Phenolic Antioxidants from Chickpeas Prevent Cytotoxicity in Human Hepatoma HuH-7 Cells Induced by Peroxyl Radicals. Antioxidants.

[6] Gao Y., Ping H., Li B.R., Li Y., Zhao F., Ma Z.H. (2021). Characterization of free, conjugated, and bound phenolics in early and late ripening kiwifruit cultivars. J. Sci. Food Agric..

[7] You B., Yang S., Yu J., Xian W., Deng Y., Huang W., Li W., Yang R. (2021). Effect of thermal and dry salt-curing processing on free and bound phenolics and antioxidant activity in Prunus mume fruits together with the phenolic bioaccessibility. LWT-Food Sci. Technol..

[8] Goyeneche R., Roura S., Ponce A., Vega-Gálvez A., Quispe-Fuentes I., Uribe E., Di Scala K. (2019). Chemical characterization and antioxidant capacity of red radish (*Raphanus sativus* L.) leaves and roots. J. Funct. Foods.

[9] Zhang J., Wang Y.D., Xue Q.W., Zhao T.R., Khan A., Wang Y.F., Liu Y.-P., Cao J.-X., Cheng G.G. (2022). The effect of ultra-high pretreatment on free, esterified and insoluble-bound phenolics from mango leaves and their antioxidant and cytoprotective activities. Food Chem..

[10] Martin-Garcia B., Gomez-Caravaca A.M., Marconi E., Verardo V. (2021). Distribution of free and bound phenolic compounds, and alkylresorcinols in wheat aleurone enriched fractions. Food Res. Int..

[11] Antognoni F., Potente G., Biondi S., Mandrioli R., Marincich L., Ruiz K.B. (2021). Free and Conjugated Phenolic Profiles and Antioxidant Activity in Quinoa Seeds and Their Relationship with Genotype and Environment. Plants.

[12] Rocchetti G., Chiodelli G., Giuberti G., Lucini L. (2018). Bioaccessibility of phenolic compounds following in vitro large intestine fermentation of nuts for human consumption. Food Chem..

[13] Persic M., Mikulic-Petkovsek M., Halbwirth H., Solar A., Veberic R., Slatnar A. (2018). Red Walnut: Characterization of the Phenolic Profiles, Activities and Gene Expression of Selected Enzymes Related to the Phenylpropanoid Pathway in Pellicle during Walnut Development. J. Agric. Food Chem..

[14] Ma Y., Kosinska-Cagnazzo A., Kerr W.L., Amarowicz R., Swanson R.B., Pegg R.B. (2014). Separation and Characterization of Soluble Esterified and Glycoside-Bound Phenolic Compounds in Dry-Blanched Peanut Skins by Liquid Chromatography-Electrospray Ionization Mass Spectrometry. J. Agric. Food Chem..

[15] Slatnar A., Mikulic-Petkovsek M., Stampar F., Veberic R., Solar A. (2015). Identification and quantification of phenolic compounds in kernels, oil and bagasse pellets of common walnut (*Juglans regia* L.). Food Res. Int..

[16] Das P.R., Islam M.T., Lee S.H., Lee M.K., Kim J.B., Eun J.B. (2020). UPLC-DAD-QToF/MS analysis of green tea phenolic metabolites in their free, esterified, glycosylated, and cell wall-bound forms by ultra-sonication, agitation, and conventional extraction techniques. Lwt-Food Sci. Technol..

[17] Prakash O., Baskaran R., Kudachikar V.B. (2019). Characterization, quantification of free, esterified and bound phenolics in Kainth (Pyrus pashia Buch.-Ham. Ex D.Don) fruit pulp by UPLC-ESI-HRMS/MS and evaluation of their antioxidant activity. Food Chem..

[18] Giambanelli E., Gómez-Caravaca A.M., Ruiz-Torralba A., Guerra-Hernández E.J., Figueroa-Hurtado J.G., García-Villanova B., Verardo V. (2020). New Advances in the Determination of Free and Bound Phenolic Compounds of Banana Passion Fruit Pulp (Passiflora tripartita, var. Mollissima (Kunth) LH Bailey) and Their In Vitro Antioxidant and Hypoglycemic Capacities. Antioxidants.

[19] Podio N.S., Baroni M.V., Wunderlin D.A. (2017). Relation between polyphenol profile and antioxidant capacity of different Argentinean wheat varieties. A Boosted Regression Trees study. Food Chem..

[20] Gong E.S., Li B., Li B., Podio N.S., Chen H., Li T., Sun X., Gao N., Wu W., Yang T. (2021). Identification of key phenolic compounds responsible for antioxidant activities of free and bound fractions of blackberry varieties extracts by boosted regression trees. J. Sci. Food Agric..

[21] Khanh-Van H., Roy A., Foote S., Vo P.H., Lall N., Lin C.-H. (2020). Profiling Anticancer and Antioxidant Activities of Phenolic Compounds Present in Black Walnuts (*Juglans nigra*) Using a High-Throughput Screening Approach. Molecules.

[22] Singleton V.L., Orthofer R., Lamuela-Raventos R.M., Packer L. (1999). Analysis of total phenols and other oxidation substrates and antioxidants by means of Folin-Ciocalteu reagent. Oxidants and Antioxidants, Pt A.

[23] Hazli U., Abdul-Aziz A., Mat-Junit S., Chee C.F., Kong K.W. (2019). Solid-liquid extraction of bioactive compounds with antioxidant potential from Alternanthera sesillis (red) and identification of the polyphenols using UHPLC-QqQ-MS/MS. Food Res. Int..

[24] Podio N.S., Lopez-Froilan R., Ramirez-Moreno E., Bertrand L., Baroni M.V., Perez-Rodriguez M.L., Sánchez-Mata M.-C., Wunderlin D.A. (2015). Matching in Vitro Bioaccessibility of Polyphenols and Antioxidant Capacity of Soluble Coffee by Boosted Regression Trees. J. Agric. Food Chem..

[25] John J.A., Shahidi F. (2010). Phenolic compounds and antioxidant activity of Brazil nut (*Bertholletia excelsa*). J. Funct. Foods.

[26] Grace M.H., Esposito D., Timmers M.A., Xiong J., Yousef G., Komarnytsky S., Lila M.A. (2016). In vitro lipolytic, antioxidant and anti-inflammatory activities of roasted pistachio kernel and skin constituents. Food Funct..

[27] de Camargo A.C., Regitano-d’Arce M.A.B., Gallo C.R., Shahidi F. (2015). Gamma-irradiation induced changes in microbiological status, phenolic profile and antioxidant activity of peanut skin. J. Funct. Foods.

[28] Jahanban-Esfahlan A., Ostadrahimi A., Tabibiazar M., Amarowicz R. (2019). A Comparative Review on the Extraction, Antioxidant Content and Antioxidant Potential of Different Parts of Walnut (*Juglans regia* L.) Fruit and Tree. Molecules.

[29] Pycia K., Kapusta I., Jaworska G., Jankowska A. (2018). Antioxidant properties, profile of polyphenolic compounds and tocopherol content in various walnut (*Juglans regia* L.) varieties. Eur. Food Res. Technol..

[30] Banc R., Rusu M.E., Filip L., Popa D.-S. (2023). The Impact of Ellagitannins and Their Metabolites through Gut Microbiome on the Gut Health and BrainWellness within the Gut. Foods.

[31] Paiva L., Rego C., Lima E., Marcone M., Baptista J. (2021). Comparative Analysis of the Polyphenols, Caffeine, and Antioxidant Activities of Green Tea, White Tea, and Flowers from Azorean Camellia sinensis Varieties Affected by Different Harvested and Processing Conditions. Antioxidants.

