# Supplementary material for: Identification of Key Antioxidants of Free, Esterified, and Bound Phenolics in Walnut Kernel and Skin
Source: Foods. 2023 Feb 15;12(4):825. doi: 10.3390/foods12040825 (PMC9956992; doi:10.3390/foods12040825)

Table S1 Mass spectrometry parameters of 37 phenolic compounds for multiple reactions monitoring (MRM)

Compounds	CAS number	Retention time (min)	Qualitative ions (m/z)	LOD (μg/g)	LOQ (μg/g)	Polarity
Ellagic acid	476-66-4	1.755	301.2/257*, 301.22/271	0.142	0.474	Negative
Gallic acid	149-91-7	0.939	168.99/125*,168.99/79.1	0.1755	0.585	Negative
Ferulic acid	1135-24-6	1.944	192.99/134.1*, 192.99/178.1	0.0458	0.153	Negative
Caffeic acid	331-39-5	1.357	178.99/135.1*	0.141	0.471	Negative
Sinapic acid	530-59-6	1.916	222.99/208.1*, 222.99/149.1	0.0244	0.0815	Negative
Vanillic acid	121-34-6	1.876	169.01/93.1*, 169.01/65.2	1.76	5.88	Positive
Syringic acid	530-57-4	1.464	196.99/182.1*, 196.99/123	0.246	0.820	Negative
Protocatechuic acid	99-50-3	1.220	155.01/93*, 155.01/65.2	1.36	4.55	Positive
Cinnamic acid	500-05-5	6.081	149.01/131.1*, 149.01/103.1	0.484	1.62	Positive
p-Hydroxybenzoic acid	99-96-7	1.648	176.96/133*, 176.96/57.1	2.50	8.33	Positive
Chlorogenic acid	327-97-9	1.426	355.01/163*, 180.9/145.1	0.0207	0.0689	Positive
p-Coumaric acid	501-98-4	1.076	138.99/95.1*,138.99/51.2	0.0156	0.0522	Negative
(+)-Catechin	7295-85-4	1.453	291.01/139*, 291.01/123	0.0917	0.306	Positive
Epicatechin	490-46-0	1.823	291.01/139*, 291.01/123	0.0608	0.203	Positive

(-)-gallocatechin	3371-27-5	0.762	307.01/139*, 307.01/151.1	0.00974	0.0325	Positive
Epigallocatechin gallate	989-51-5	1.794	459.01/139*, 459.01/289.1	0.00487	0.0162	Positive
(-)-Gallocatechin gallate	4233-96-9	1.912	459/139*, 459/289.1	0.00224	0.00746	Positive
(-)-Epicatechin gallate	1257-08-5	2.342	443.01/123.1*, 443.01/139.1	0.0524	0.175	Positive
(-)-Epigallocatechin	970-74-1	1.208	307.01/139*, 307.01/151.1	0.0147	0.0492	Positive
Catechin gallate	130405-40-2	2.710	443.01/123.1*, 443.01/139.1	0.00078	0.00260	Positive
Rutin	153-18-4	2.690	611.01/303.1*, 611.01/465.1	0.00376	0.0126	Positive
Quercetin-3-o-rutinose	949926-49-2	2.405	611.01/287.1*, 611.01/449.2	0.00145	0.00484	Positive
Quercetin-7-O-β-D-glucopyranoside	491-50-9	2.834	465.39/303.1*	0.0162	0.0539	Positive
Quercetin-3-O-glucopyranoside	482-35-9	2.909	465.01/303.1*, 465.01/85.1	0.0133	0.0444	Positive
Quercetin	117-39-5	3.383	301.23/151*, 301.23/179.1	0.00624	0.0208	Negative
Dihydroquercetin	480-18-2	2.973	305.3/259.1*, 305.3/153	0.0217	0.0722	Positive
Dihydrokaempferol	480-20-6	3.966	289.26/153*, 289.26/215.1	0.00891	0.0297	Positive
Kaempferol	520-18-3	3.600	287.01/153.1*, 287.01/121	0.00671	0.0224	Positive
Kaempferol-3-O-glucosylside	480-10-4	3.604	449.39/287.1*, 449.39/89.1	0.00557	0.0186	Positive
Naringenin	67604-48-2	6.902	273.26/153*, 273.26/115.1	0.00752	0.0251	Positive
Vitexin	3681-93-4	2.728	433.01/313.1*, 433.01/415.1	0.00404	0.0135	Positive

Procyanidin B2	29106-49-8	1.515	579.53/127,579.53/291	0.0338	0.113	Positive
juglone	481-39-0	5.626	173.14/145*	0.0915	0.305	Negative
Dendrobine	2115-91-5	0.967	286.37/226*,286.37/250	0.0349	0.116	Positive
Cumallic acid	500-05-0	1.074	139.08/95,139.08/51.2	0.0168	0.0559	Negative
Lycorine	476-28-8	0.762	288.37/176.9,288.37/147	0.00057	0.00188	Positive
Luteolin	491-70-3	5.803	287.25/153*, 287.25/135.1	0.0137	0.0457	Positive

* quantitative ion

Figure S1. Total ions chromatograph (TIC) obtained at negative (A) and positive (B) MS ionization for standards of phenolic compounds

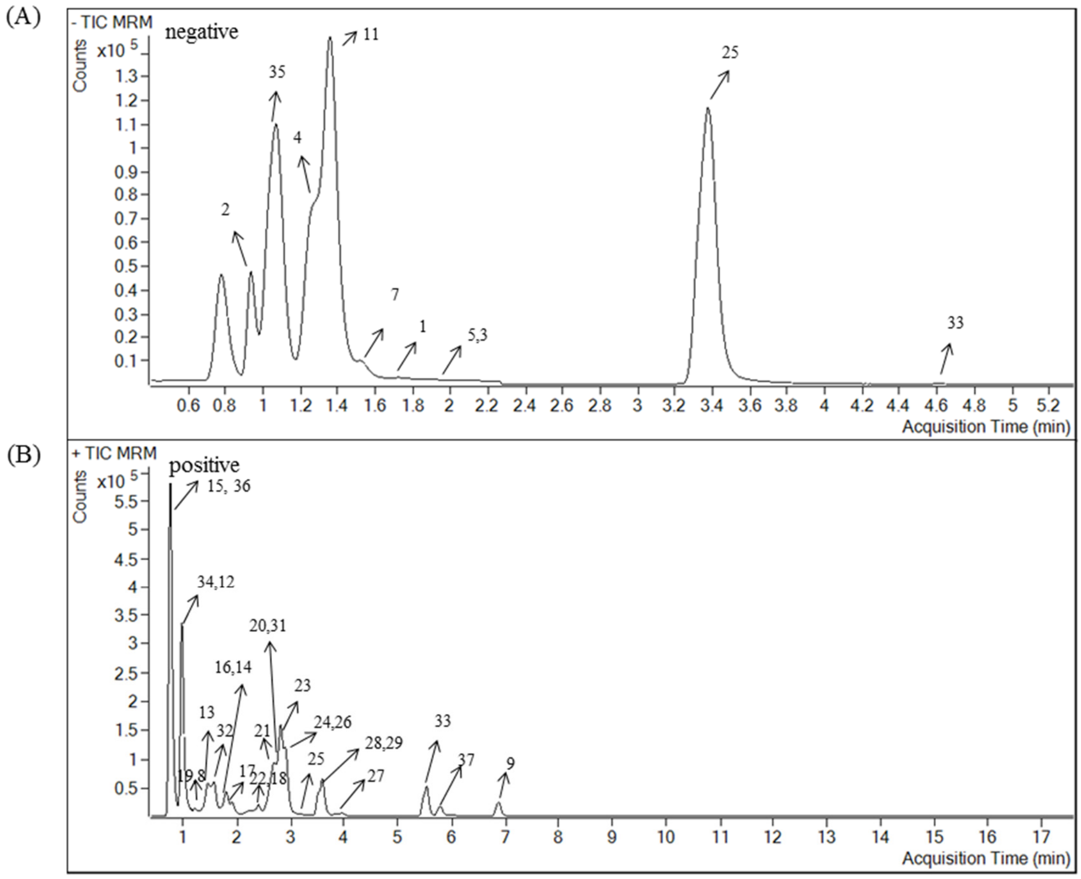

1 Ellagic acid, 2 Gallic acid, 3 Ferulic acid, 4 Caffeic acid, 5 Sinapic acid, 6 Vanillic acid, 7 Syringic acid, 8 Protocatechuic acid, 9 Cinnamic acid, 10 *p*-Hydroxybenzoic acid, 11 Chlorogenic acid, 12 *p*-Coumaric acid, 13 (+)-Catechin, 14 Epicatechin, 15 (-)-gallocatechin, 16 Epigallocatechin gallate, 17 (-)-Gallocatechin gallate, 18 (-)-Epicatechin gallate, 19 (-)-Epigallocatechin , 20 Catechin gallate, 21 Rutin, 22 Quercetin-3-o-rutinose, 23 Quercetin-7-O-β-D-glucopyranoside, 24 Quercetin-3-O-glucopyranoside, 25 Quercetin, 26 Dihydroquercetin, 27 Dihydrokaempferol, 28 Kaempferol, 29 Kaempferol-3-O-glucosylside, 30 Naringenin, 31 Vitexin, 32 Procyanidin B2, 33 juglone, 34 Dendrobine, 35 Cumallic acid, 36 Lycorine, 37 Luteolin

Figure S2. Typical chromatograms obtained at negative (A) and positive (B) MS ionization for standards of phenolic compounds

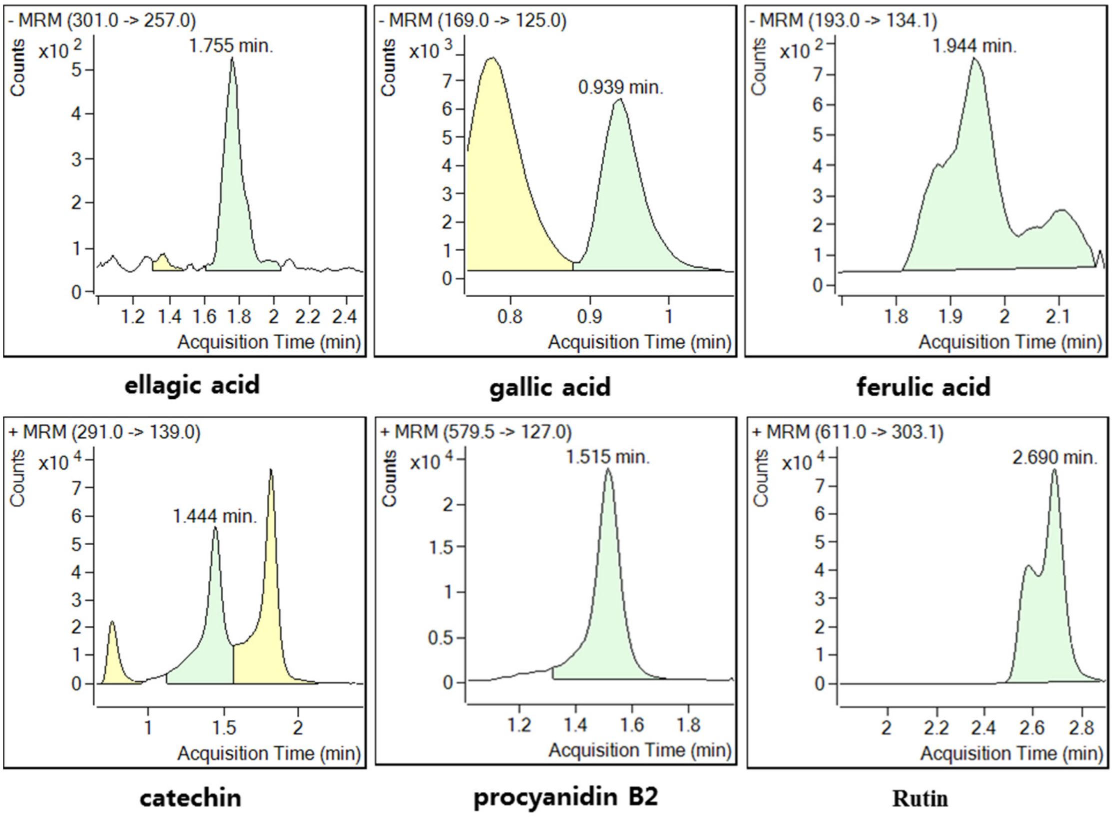

Supplement: Supplementary file 1 [file foods-12-00825-s001.zip › foods-2185504-supplementary.pdf]
